# Alfalfa fields with different stand ages alters soil inorganic nitrogen dynamics through shifts in nitrogen-cycling functional genes across growth stages

**DOI:** 10.3389/fmicb.2026.1958461

**Published:** 2026-10-02

**Authors:** Delong Tian, Mingzi Lv, Guoshuai Wang, Ting Fan

**Affiliations:** 1 Institute of Pastoral Hydraulic Research, Ministry of Water Resources (IPHR-MWR), Hohhot, China; 2 Yinshanbeilu Grassland Eco-Hydrology National Observation and Research Station, China Institute of Water Resources and Hydropower Research, Beijing, China

**Keywords:** alfalfa, cultivation duration, microbial nitrogen cycling, nitrogen-cycling functional genes, soil inorganic nitrogen, structural equation modeling

## Abstract

**Introduction:**

Alfalfa cultivation is associated with soil nitrogen availability and microbial nitrogen-cycling functional potential. However, how soil inorganic nitrogen and nitrogen-cycling functional genes vary with alfalfa stand age across growth stages remains unclear.

**Methods:**

Using a chronosequence approach, we investigated soil inorganic nitrogen and nitrogen-cycling functional genes in a maize-field control (CK) and 2-, 3-, 4-, and 7-year-old alfalfa fields (T1–T4) during the regreening, flowering, and maturity-harvest stages. Soil ${\text{NO}}_{3}^{-}$-N and ${\text{NH}}_{4}^{+}$-N concentrations and TPM-normalized abundances of nitrogen-cycling functional genes were determined. Correlation analysis, PERMANOVA, and structural equation modeling (SEM) were used to examine their relationships.

**Results:**

Soil ${\text{NO}}_{3}^{-}$-N and ${\text{NO}}_{4}^{+}$-N concentrations differed significantly among stand ages and generally exhibited unimodal patterns, with the highest observed values in the 4-year field (T3) and lower values in the 7-year field (T4). The abundances of genes involved in nitrification, denitrification, and nitrate reduction also varied with stand age and showed growth-stage-specific patterns. Growth stage explained a greater proportion of variation in nitrogen-cycling gene profiles than stand age, although stand age was also significantly associated with these profiles. The relationships between functional genes and soil inorganic nitrogen varied among growth stages. SEM analysis suggested that growth stage was the primary factor associated with variation in nitrogen-cycling gene groups. Nitrification-related genes were positively correlated with soil inorganic nitrogen, whereas nitrate reduction-related genes showed negative correlations.

**Conclusion:**

Overall, the 4-year alfalfa field exhibited the highest observed soil inorganic nitrogen concentrations, while this pattern was less evident in the 7-year field. These findings indicate that soil nitrogen status and nitrogen-cycling functional genes vary jointly with alfalfa stand age and growth stage. Nitrogen-cycling functional genes may serve as potential indicators of soil nitrogen status, although further experimental studies are needed to verify these relationships.

## 1 Introduction

Soil nitrogen cycling is a fundamental biogeochemical process that sustains ecosystem productivity and soil quality in agricultural and grassland ecosystems. Soil inorganic nitrogen forms, including ammonium nitrogen (${\text{NO}}_{4}^{+}$-N) and nitrate nitrogen (${\text{NO}}_{3}^{-}$-N), together with microbial biomass nitrogen (MBN), not only reflect soil nitrogen availability but are also closely associated with key nitrogen transformation processes, including nitrogen mineralization, nitrification, denitrification, and microbial nitrogen immobilization (Yan et al., 2017; Gao et al., 2019; Liu et al., 2023). Alfalfa (*Medicago sativa* L.), an important perennial leguminous forage crop in China, exhibits strong biological nitrogen fixation capacity. Its extensive root system and substantial belowground biomass can modify soil nitrogen accumulation and transformation processes through multiple pathways, including symbiotic nitrogen fixation by root nodules, rhizodeposition, and the return of plant residues (Zhao et al., 2020; Xu et al., 2023; Li Q. P. et al., 2024). Previous studies have demonstrated that alfalfa cultivation can increase soil nitrogen and organic matter contents and reshape rhizosphere bacterial and fungal community structures, highlighting the important role of alfalfa in influencing soil nutrient availability and microbial processes (Qi et al., 2023). Therefore, investigating the effects of different alfalfa cultivation durations on soil nitrogen pools and transformation processes is essential for evaluating changes in soil fertility and determining appropriate cultivation durations for sustainable alfalfa production.

Previous studies have shown that alfalfa cultivation substantially affects soil nitrogen availability and nitrogen cycling processes. On the one hand, biological nitrogen fixation by alfalfa root nodules and the incorporation of plant residues provide additional nitrogen inputs to soils, thereby increasing soil total nitrogen, available nitrogen, and microbial biomass nitrogen levels. On the other hand, prolonged cultivation duration can alter plant growth status, root distribution patterns, residue inputs, and soil moisture and nutrient conditions, resulting in pronounced duration-dependent changes in soil nitrogen accumulation (Zhang et al., 2019; Xu et al., 2023; Liu et al., 2025). Some studies have suggested that extending alfalfa cultivation duration promotes soil nutrient accumulation and improves soil quality, whereas others have reported that long-term monoculture may lead to excessive soil water consumption, nutrient imbalance, or continuous cropping constraints, thereby reducing the soil improvement effects of alfalfa (Zheng et al., 2017; Wang et al., 2025; Kang et al., 2024; Zhang et al., 2024). Consequently, the cultivation duration associated with favorable outcomes may vary among soil types, climatic conditions, and management practices, and the responses of soil nitrogen dynamics to cultivation duration require further evaluation under specific environmental conditions. Recent studies have further revealed that alfalfa cultivation duration significantly affects the composition of ammonia-oxidizing microbial communities and the abundances of functional genes involved in nitrification, suggesting that changes in cultivation duration may influence soil nitrogen transformation through alterations in nitrogen-cycling microbial processes (Li L. et al., 2025).

Soil nitrogen transformation is influenced not only by the availability of nitrogen substrates but also by is also closely associated with microbial community composition and functional potential (Wang et al., 2018; Geng et al., 2020; Yin et al., 2026). Soil microorganisms participate in key nitrogen cycling processes, including nitrification, denitrification, nitrate reduction, and nitrogen fixation, through nitrogen-cycling functional genes. These functional genes serve as indicators of the microbial potential involved in soil nitrogen transformation processes (Wang et al., 2023; Sun et al., 2023; Xu et al., 2023; Saghaï et al., 2023; Xie et al., 2023; Xing et al., 2022). Therefore, integrating analyses of soil nitrogen pools, microbial community composition, and nitrogen-cycling functional genes can provide insights into the potential mechanisms underlying the effects of alfalfa cultivation duration on soil nitrogen cycling. Recent studies on alfalfa-associated microbial communities have demonstrated that different alfalfa cultivars, salinity conditions, and regional environmental gradients can significantly alter bacterial and fungal community structures and their functional potentials in rhizosphere and root-associated soils (Chen et al., 2025; Zhou et al., 2026; Lv et al., 2026; Xu et al., 2025). These findings provide a microbial perspective for understanding changes in soil nitrogen cycling processes in alfalfa fields.

The Tumochuan Plain is a typical agro-pastoral ecotone and an important forage production region, where alfalfa cultivation is widely practiced. However, soil salinization and limitations in water and nutrient availability are major constraints in this region. Fertilization and soil improvement practices can substantially influence soil physicochemical properties, enzyme activities, and microbial community structures (Li H. X. et al., 2025; Duan et al., 2025; Wang et al., 2026). Studies conducted in saline-alkali soils have shown that alfalfa cultivation can improve soil pore structure, enhance microbial diversity, increase soil organic carbon content, and promote the restoration of soil fertility (Zhu et al., 2025). Nevertheless, under long-term monoculture conditions and regional water and nutrient limitations, how soil inorganic nitrogen accumulation, microbial community composition, and nitrogen-cycling functional genes respond to prolonged alfalfa cultivation remains unclear.

Based on these considerations, we hypothesized that different durations of alfalfa cultivation would induce variations in soil inorganic nitrogen concentrations, microbial community composition, and the abundances of nitrogen-cycling functional genes, thereby influencing soil nitrogen transformation processes through changes in microbial functional potential. To test this hypothesis, this study investigated the variations in soil ${\text{NO}}_{4}^{+}$-N and ${\text{NO}}_{3}^{-}$-N concentrations, microbial community composition, and nitrogen-cycling functional gene abundances across different alfalfa cultivation durations. Correlation analysis and structural equation modeling (SEM) were further applied to explore the relationships between soil inorganic nitrogen dynamics and nitrogen-cycling functional gene responses. This study provides theoretical insights for determining appropriate alfalfa cultivation durations and improving soil nitrogen management in the Tumochuan Plain.

## 2 Materials and methods

### 2.1 Study area and experimental design

#### 2.1.1 Description of the study area

The field experiment was conducted from April to September 2025 in Gouziban Village, Bikedqi Town, Tumed Left Banner, Hohhot, Inner Mongolia Autonomous Region, China (40.6908°N, 111.3377°E). The study area is located in the Tumochuan Plain and experiences a temperate continental monsoon climate characterized by cold and prolonged winters, dry and windy springs, concentrated precipitation in summer, and cool and short autumns. The mean annual temperature is 7.2 °C, with average temperatures of −11.3 °C in January and 22.8 °C in July. The recorded extreme maximum and minimum temperatures are 37.2 °C and −35.6 °C, respectively. The region has an average frost-free period of 133 days, a maximum soil freezing depth of 131 cm, an annual sunshine duration of 2,952.1 h, and an annual total solar radiation of 133.82 kJ cm^−2^. The mean annual precipitation is 379.4 mm, with most precipitation occurring from June to August (Figure 1).

**Figure 1:**
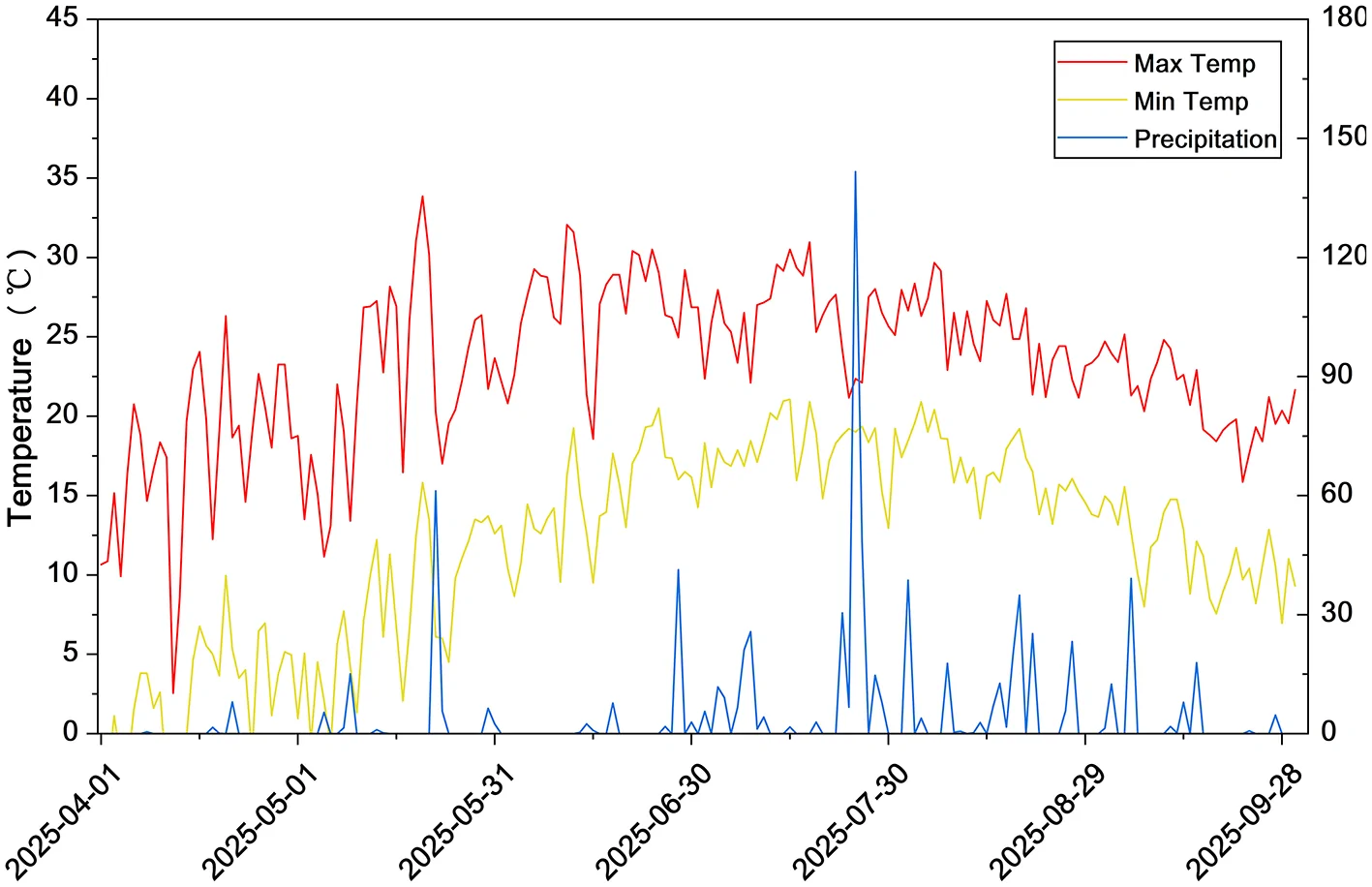
Monthly variations in maximum temperature, minimum temperature, and precipitation in the experimental area during 2025.

The study area is an important forage production region in Hohhot, where alfalfa cultivation is widely distributed. The soil type in the experimental area is classified as cinnamon sandy soil, characterized by a sandy texture and weak alkalinity. The soil bulk density in the 0–20 cm layer was 1.47 g cm^−3^. The selected fields had the same soil type and similar topographic conditions, irrigation regime, and management practices. However, because the alfalfa fields were established in different years, baseline soil nutrient properties and field history may have differed among stand ages. This potential limitation was considered when interpreting the effects of alfalfa stand age.

#### 2.1.2 Experimental design

This study used an observational chronosequence, or space-for-time substitution, approach to assess the associations between alfalfa stand age, soil inorganic nitrogen, and microbial nitrogen-cycling functional genes. The experimental fields were existing alfalfa fields established in different years rather than plots randomly assigned to different cultivation durations within the same year. Four alfalfa stand-age groups were selected according to the year of establishment: T1, 2-year-old alfalfa sown in 2023; T2, 3-year-old alfalfa sown in 2022; T3, 4-year-old alfalfa sown in 2021; and T4, 7-year-old alfalfa sown in 2018. A maize field without alfalfa cultivation was included as the control (CK). Thus, CK represented a maize-field control rather than an uncultivated field.

For each treatment group, three replicate sampling plots were established within the corresponding field. Each treatment field covered approximately 0.67 ha. The fields were adjacent to each other and separated by approximately 2–3 m buffer strips or field paths. They were selected to have similar soil types, topographic conditions, irrigation regimes, and management practices. Alfalfa stand age was treated as a grouping factor rather than a randomly assigned experimental factor.

The alfalfa cultivar used in this experiment was “Algonquin.” Seeds were sown at a depth of 2 cm with a row spacing of 15 cm, and the seeding rate was 22.5 kg ha^−1^. Liquid diammonium phosphate fertilizer was applied during the annual regreening stage at a rate of 500 kg ha^−1^. Drip irrigation was applied during the first cultivation year to ensure seedling establishment, whereas sprinkler irrigation was used in subsequent years. Irrigation scheduling followed local long-term production practices, with three irrigation events applied annually and a total irrigation amount of 240 mm. The first irrigation event was conducted around April 20 with 90 mm of water, followed by the second event around June 20 with 60 mm and the third event around July 30 with 90 mm.

All alfalfa plots were established in breeding alfalfa fields. Unlike forage-production fields, which are commonly harvested several times per year, these breeding alfalfa fields were managed for seed production and breeding evaluation. Therefore, alfalfa was retained until seed maturity and harvested once annually during the seed maturity stage, consistent with local management practices for breeding alfalfa fields.

### 2.2 Soil sampling

Rhizosphere soil samples were collected during three alfalfa growth stages in 2025: the regreening stage (mid-April), flowering stage (early July), and maturity-harvest stage (late September). A five-point sampling method was used to collect rhizosphere soil samples from the 0–20 cm soil layer in alfalfa fields with different cultivation durations. Before sampling, surface debris, dead plant residues, and gravel were removed. The alfalfa plants were carefully excavated, and the roots were shaken to remove loosely adhering soil. Soil remaining attached to the roots was collected as rhizosphere soil and passed through a 2-mm sieve to remove root fragments and visible plant residues. Root nodules were not separately collected or analyzed in this study; therefore, their contribution to the rhizosphere microbial and functional gene profiles could not be evaluated separately.

Collected rhizosphere soil samples were divided into two portions. One portion was placed into 25 mL centrifuge tubes, immediately preserved on dry ice, and transported for metagenomic sequencing. The other portion was stored in sealed plastic bags for the determination of soil ammonium nitrogen (${\text{NO}}_{4}^{+}$-N) and nitrate nitrogen (${\text{NO}}_{3}^{-}$-N) concentrations.

### 2.3 Determination of soil properties

#### 2.3.1 Determination of soil inorganic nitrogen

Soil ammonium nitrogen (${\text{NO}}_{4}^{+}$-N) concentration was extracted using potassium chloride (KCl) solution and determined using the indophenol blue colorimetric method according to the Chinese forestry industry standard (State Forestry Administration of the People's Republic of China, 2015). Soil nitrate nitrogen (${\text{NO}}_{3}^{-}$-N) concentration was extracted using KCl solution and quantified using the dual-wavelength colorimetric method following the Chinese environmental protection standard (Ministry of Environmental Protection of the People's Republic of China, 2012).

#### 2.3.2 Microbial community profiling and nitrogen metabolism functional analysis

Total microbial DNA was extracted from rhizosphere soil samples using the E.Z.N.A.^®^ Stool DNA Kit. Metagenomic library construction and sequencing were performed by Shanghai Lingen Biotechnology Co., Ltd. Genomic DNA was randomly fragmented using a Covaris S220 ultrasonicator, and DNA fragments of approximately 450 bp were selected for library preparation. After quality assessment, the qualified libraries were sequenced on the Illumina NovaSeq 6000 platform using a paired-end 150 bp sequencing strategy.

Raw sequencing reads were subjected to quality control using Trimmomatic to remove adapter sequences, contaminant sequences, and low-quality reads. Host-derived reads were removed by mapping the quality-controlled reads against the alfalfa (*Medicago sativa* L.) reference genome using BWA mem with the parameters “-M -k 32 -t 16”. Reads that did not map to the host genome were retained as clean non-host reads for downstream analyses. The raw sequencing data contained 67.58–79.71 million reads per sample. After quality filtering and host removal, 62.19–73.38 million clean reads were obtained per sample, with Q20 values of 97.84–98.18%, Q30 values of 93.88–94.62%, and GC contents of 62.56–63.83%. The sequencing depth, quality-control statistics, and host-removal statistics for each sample are provided in Supplementary Table S2. Taxonomic classification of clean reads was performed using Kraken2 (Wood and Salzberg, 2014), and Bracken was applied to estimate species relative abundances at different taxonomic levels for subsequent microbial community composition analyses. Metagenomic assembly was performed using MEGAHIT (Li et al., 2015) based on the clean non-host reads, with a minimum contig length of 500 bp using the parameter “–min-contig-len 500”. Assembly statistics, including the number of contigs, total assembly length, N50, and N90, are provided in Supplementary Table S3. Open reading frames (ORFs) were predicted from the assembled contigs using Prodigal, and the predicted genes were clustered and dereplicated using CD-HIT (Fu et al., 2012) to construct a non-redundant gene catalog. Gene abundance was quantified by mapping the clean non-host reads from each sample against the non-redundant gene catalog using Salmon (Patro et al., 2017).

To account for differences in sequencing depth and gene length among samples, gene abundance was normalized and expressed as transcripts per million (TPM). TPM was calculated as follows:


$$\begin{array}{l} TPM=\frac{N_{g}}{L_{g}}\times \frac{1}{\sum j\frac{N_{j}}{L_{j}}}\times 10^{6} \end{array}$$

where *N*_*g*_ represents the average number of reads mapped to gene g, *L*_*g*_ represents the length of gene g, and j represents all genes in the non-redundant gene catalog. Therefore, the functional gene abundance values reported in this study represent metagenome-derived TPM-normalized relative abundances rather than absolute gene copy numbers.

Nitrogen-cycling-related functional genes were identified based on functional annotations associated with nitrogen metabolism. Functional annotation of nitrogen-cycling genes was performed against the NCycDB database using DIAMOND, with an e-value threshold of 1 × 10^−5^ and a minimum sequence identity of 70%. The analyzed genes included those related to nitrification, denitrification, nitrate transformation, and nitrogen fixation. Specifically, amoA (K10944) and hao (K10535) were assigned to nitrification; nirK (K00368), nirS (K15864), norB (K04561), and nosZ (K00376) were assigned to denitrification; napA (K02567), nirB (K00362), nrfA (K03385), and nasA (K00372) were assigned to nitrate reduction; and nifH was assigned to nitrogen fixation based on the NCycDB annotation subtype. The annotation of narG/nxrA corresponded to K00370, which is annotated in KEGG as “narG, narZ, nxrA; nitrate reductase / nitrite oxidoreductase, alpha subunit”. Because narG and nxrA are homologous genes and may represent different nitrate or nitrite transformation processes, narG/nxrA was treated as a nitrate-transformation-related homolog group rather than a pathway-specific marker gene. A functional gene was considered detected when it had a qualified database annotation and a TPM-normalized abundance greater than zero. Under these criteria, nifH was detected only in the regreening-stage samples and was not detected in the flowering- or maturity-harvest-stage samples. Therefore, nifH non-detection was interpreted as the absence of a qualified annotation under the applied sequencing and annotation criteria, rather than evidence that the gene or biological nitrogen fixation was completely absent from the soil.

### 2.4 Data processing and statistical analysis

Raw data were organized using Microsoft Excel 2019. Statistical analyses and data visualization were performed using Origin 2025 and R software (version 4.4.3). The Shapiro-Wilk test and Levene's test were conducted to assess data normality and homogeneity of variance, respectively. For datasets satisfying the assumptions of normality and homogeneity of variance, one-way analysis of variance (one-way ANOVA) was performed to evaluate differences among treatments, followed by Tukey's honestly significant difference (HSD) test for multiple comparisons. For datasets that did not meet these assumptions, the Kruskal-Wallis rank-sum test was applied. Statistical significance was determined at *p* < 0.05.

Before statistical analysis, TPM-normalized abundances of nitrogen-cycling functional genes were log10 (x + 1)-transformed. Spearman correlation analysis was performed to examine relationships between nitrogen-cycling functional genes and soil inorganic nitrogen concentrations, and correlation patterns were visualized using R packages. Mantel tests were conducted to evaluate the relationships among nitrogen-cycling functional gene profiles, soil environmental factors, and microbial community composition. Bray-Curtis distance matrices were used for distance calculations, and significance was assessed using 999 permutations.

To quantify the relative contributions of alfalfa stand age and growth stage to variation in nitrogen-cycling functional gene profiles, permutational multivariate analysis of variance (PERMANOVA) was performed using the adonis2 function in the vegan package in R. Bray–Curtis dissimilarity matrices were calculated based on log10(x + 1)-transformed TPM-normalized abundances of nitrogen-cycling functional genes. Because the CK treatment represented a maize field rather than an alfalfa stand age, PERMANOVA was conducted using only the alfalfa treatments (T1–T4). The model was specified as gene profile ~ stand age + growth stage, with stand age listed first and growth stage listed second. Marginal tests of terms were performed using by = “margin”, and the independent contribution of each factor was evaluated based on its marginal R^2^ value. Statistical significance was assessed using 999 permutations.

Data processing and visualization were primarily conducted using R packages, including dplyr, ggplot2, and vegan. Structural equation modeling (SEM) was performed as an exploratory path analysis to examine the associations among alfalfa stand age, growth stage, nitrogen-cycling functional gene groups, and soil inorganic N. The model included six observed or composite variables: alfalfa stand age, growth stage, nitrification-related genes, denitrification-related genes, nitrate reduction-related genes, and soil inorganic N.

Composite variables for nitrogen-cycling functional gene groups were constructed using the first principal component (PC1) extracted from principal component analysis (PCA) of TPM-normalized gene abundances. Specifically, nitrification-related genes included amoA and hao; denitrification-related genes included nirK, nirS, norB, and nosZ; and nitrate reduction- or assimilation-related genes included napA, nirB, nrfA, and nasA. The K00370 annotation combines narG, narZ, and nxrA homologs associated with nitrate reductase or nitrite oxidoreductase functions. Because these homologs may be involved in different nitrate/nitrite transformation processes and the K00370 annotation does not resolve the direction of the transformation, narG/nxrA was excluded from the nitrate reduction- or assimilation-related composite variable and was analyzed separately as a nitrate/nitrite-transformation-related homolog group. Soil inorganic N was represented by PC1 extracted from ${\text{NO}}_{4}^{+}$-N and ${\text{NO}}_{3}^{-}$-N concentrations. All variables were standardized before SEM analysis.

Alfalfa stand age was coded according to cultivation duration, and growth stage was coded according to the seasonal sequence of alfalfa development: regreening, flowering, and maturity-harvest stages. SEM was conducted using the lavaan package in R version 4.4.3 with maximum likelihood estimation. The sample size used for SEM was n = 45. Model fit was evaluated using χ^2^, degrees of freedom, *p* value, comparative fit index (CFI), Tucker-Lewis index (TLI), root mean square error of approximation (RMSEA), and standardized root mean square residual (SRMR). Standardized path coefficients and coefficients of determination (R^2^) were reported. Because the SEM was based on observational chronosequence data, the model was interpreted as an exploratory path model describing statistical associations rather than direct evidence of causal mechanisms.

## 3 Results

### 3.1 Effects of different durations of alfalfa cultivation on soil inorganic nitrogen distribution

As shown in Figure 2, different durations of alfalfa cultivation significantly affected soil ${\text{NO}}_{3}^{-}$-N and ${\text{NO}}_{4}^{+}$-N concentrations. Across the three growth stages, soil ${\text{NO}}_{3}^{-}$-N concentration was consistently highest in the T3 treatment, which was significantly higher than those in the CK, T1, T2, and T4 treatments, whereas no significant differences were observed among the remaining treatments. During the regreening, flowering, and maturity-harvest stages, ${\text{NO}}_{3}^{-}$-N concentrations in the T3 treatment were approximately 11.4, 12.3, and 11.8 mg kg^−1^, respectively, indicating the greatest accumulation of nitrate nitrogen under the 4-year alfalfa cultivation treatment.

**Figure 2:**
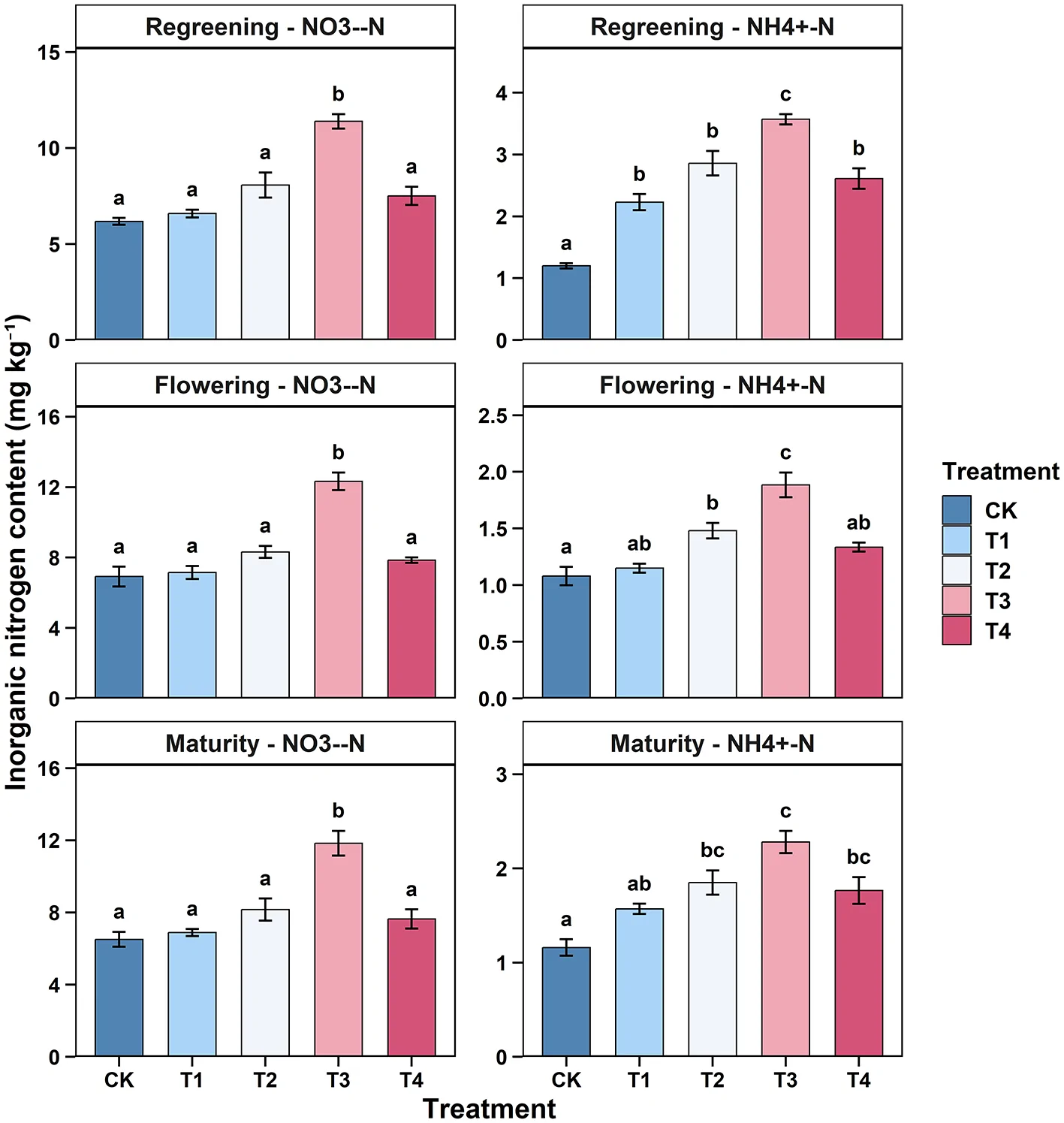
Changes in soil inorganic nitrogen concentrations under different durations of alfalfa cultivation across different growth stages. CK, maize-field control without alfalfa cultivation; T1, 2-year-old alfalfa field; T2, 3-year-old alfalfa field; T3, 4-year-old alfalfa field; T4, 7-year-old alfalfa field.

Soil ${\text{NO}}_{4}^{+}$-N concentrations also exhibited a unimodal response pattern with increasing alfalfa cultivation duration, increasing initially and subsequently declining. During the regreening stage, ${\text{NO}}_{4}^{+}$-N concentration was highest in the T3 treatment and was significantly higher than those in all other treatments. The T1, T2, and T4 treatments showed significantly higher ${\text{NO}}_{4}^{+}$-N concentrations than CK. Similar patterns were observed during the flowering and maturity-harvest stages, with the highest ${\text{NO}}_{4}^{+}$-N concentration detected in the T3 treatment, whereas the T4 treatment showed a decline compared with T3. Compared with ${\text{NO}}_{3}^{-}$-N, ${\text{NO}}_{4}^{+}$-N exhibited greater temporal variation among growth stages, with overall higher concentrations during the regreening stage than during the flowering and maturity-harvest stages.

Overall, soil inorganic nitrogen concentrations displayed a consistent pattern of initial increase followed by decline with prolonged alfalfa cultivation duration. The T3 treatment showed the highest accumulation of both ${\text{NO}}_{3}^{-}$-N and ${\text{NO}}_{4}^{+}$-N, whereas the T4 treatment exhibited reduced concentrations compared with T3, suggesting that moderate-duration alfalfa cultivation was more favorable for maintaining higher soil inorganic nitrogen availability.

### 3.2 Effects of different durations of alfalfa cultivation on nitrogen-cycling functional gene abundances

The abundances of soil nitrogen-cycling functional genes varied among treatments across different growth stages. As shown in Figure 3, during the regreening stage, significant differences among treatments were observed for most functional genes, except for nirK and nifH. Among these genes, narG/nxrA, napA, amoA, and nosZ exhibited highly significant differences among treatments (*p* < 0.001). Overall, the CK treatment showed relatively higher abundances for most functional genes, whereas amoA abundance reached the highest level in the T3 treatment. Under the applied NCycDB annotation and TPM-based detection criteria, qualified nifH annotations were detected only in the regreening-stage samples, whereas no qualified nifH annotation was detected in the flowering- or maturity-harvest-stage samples. Accordingly, nifH abundance was not reported for the latter two growth stages.

**Figure 3:**
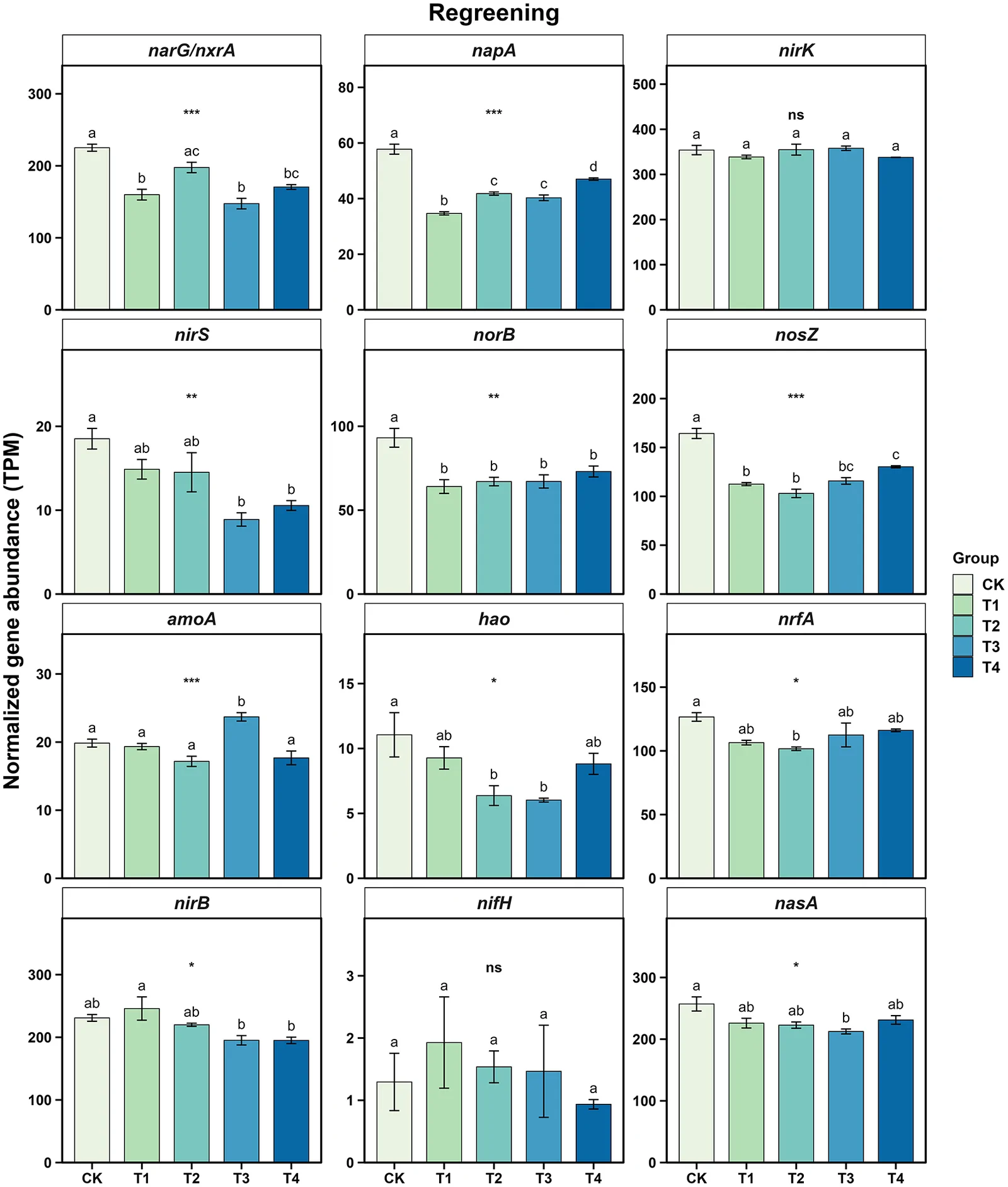
Changes in TPM-normalized abundances of soil nitrogen-cycling functional genes among different alfalfa stand ages during the regreening stage. CK, maize-field control without alfalfa cultivation; T1, 2-year-old alfalfa field; T2, 3-year-old alfalfa field; T3, 4-year-old alfalfa field; T4, 7-year-old alfalfa field. Gene abundance values represent metagenome-derived TPM-normalized relative abundances rather than absolute gene copy numbers. Data are presented as means ± SE (*n* = 3). Different lowercase letters indicate significant differences among treatments at *p* < 0.05. ns indicates no significant difference (*p* ≥ 0.05), whereas *, **, and *** indicate significance at *p* < 0.05, *p* < 0.01, and *p* < 0.001, respectively.

During the flowering stage (Figure 4), all nitrogen-cycling functional genes exhibited significant differences among treatments. Among them, narG/nxrA, napA, nirK, norB, hao, and nirB showed highly significant differences (*p* < 0.001). The CK treatment exhibited relatively higher abundances of narG/nxrA, napA, nirS, nosZ, nirB, and nasA, whereas norB, amoA, and nrfA showed relatively higher abundances in the T4 treatment.

**Figure 4:**
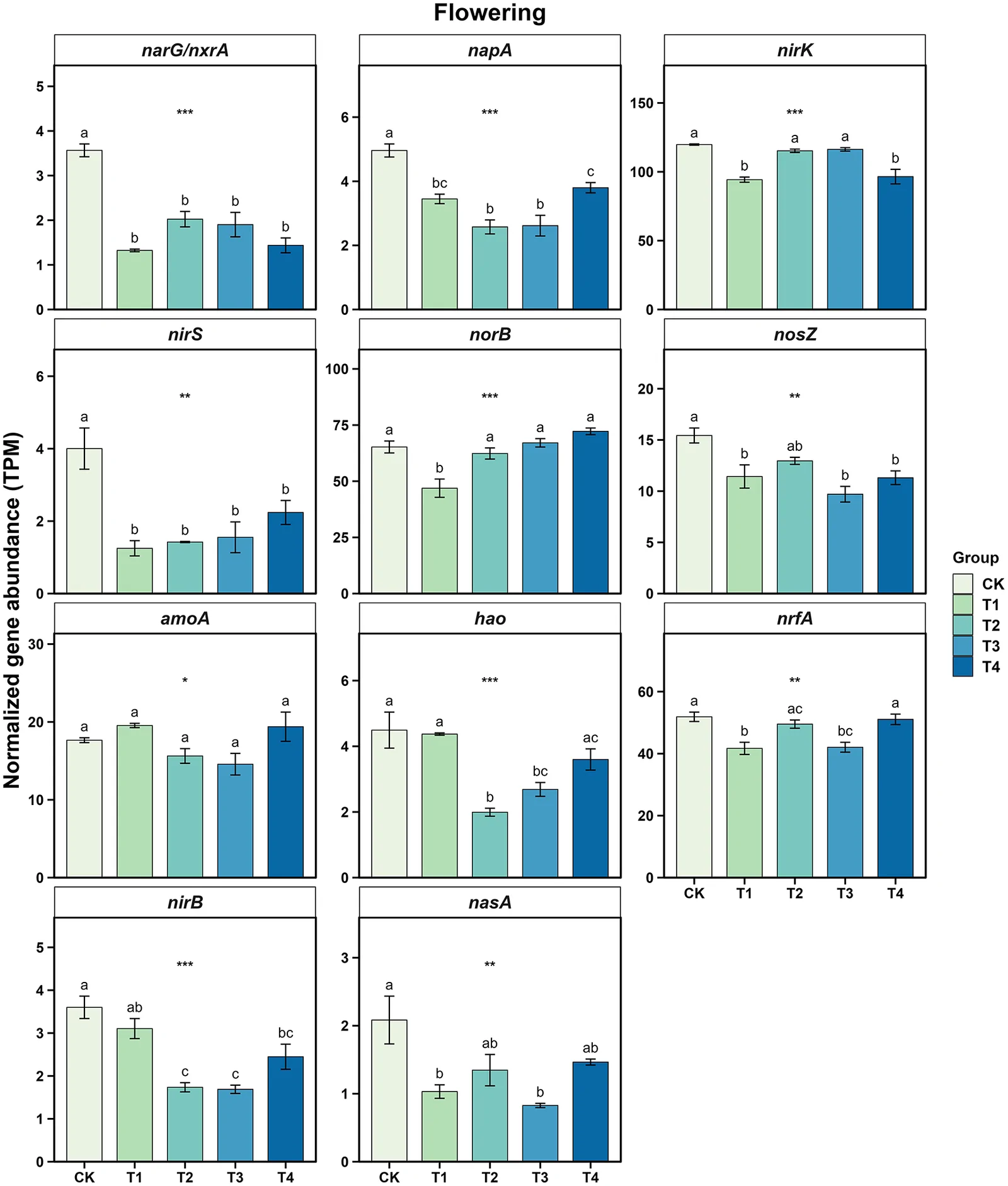
Changes in TPM-normalized abundances of soil nitrogen-cycling functional genes among different alfalfa stand ages during the flowering stage. CK, maize-field control without alfalfa cultivation; T1, 2-year-old alfalfa field; T2, 3-year-old alfalfa field; T3, 4-year-old alfalfa field; T4, 7-year-old alfalfa field. Gene abundance values represent metagenome-derived TPM-normalized relative abundances rather than absolute gene copy numbers. Data are presented as means ± SE (*n* = 3). Different lowercase letters indicate significant differences among treatments at *p* < 0.05. ns indicates no significant difference (*p* ≥ 0.05), whereas *, **, and *** indicate significance at *p* < 0.05, *p* < 0.01, and *p* < 0.001, respectively.

During the maturity-harvest stage (Figure 5), significant differences among treatments were detected for all functional genes except hao. Among these genes, napA, nirK, nirS, norB, nosZ, nrfA, nirB, and nasA showed highly significant differences (*p* < 0.001). The CK treatment exhibited higher abundances of narG/nxrA, napA, nosZ, nrfA, nirB, and nasA, whereas the T3 treatment showed higher abundances of nirK, nirS, and norB. The highest abundance of amoA was observed in the T1 treatment.

**Figure 5:**
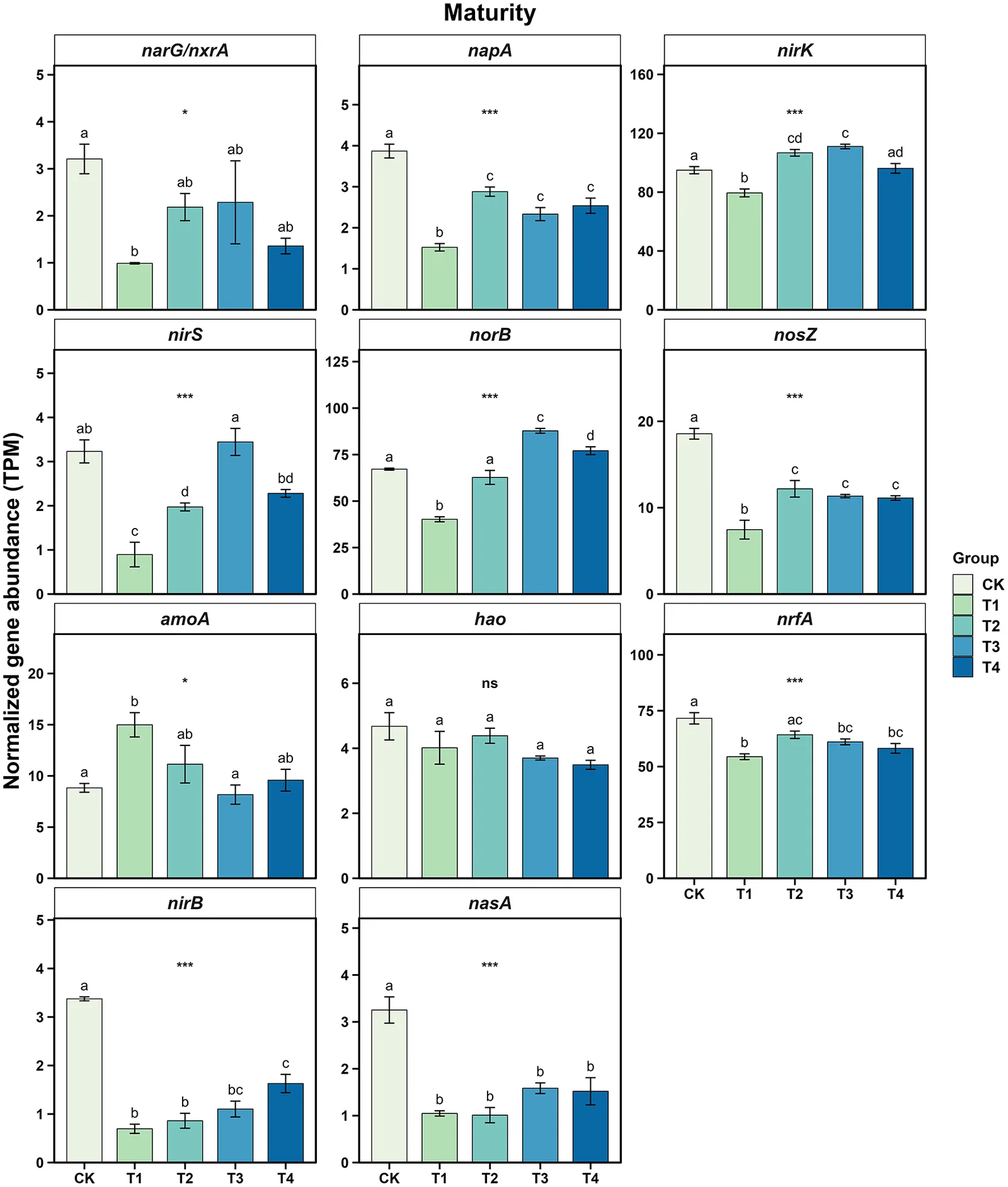
Changes in TPM-normalized abundances of soil nitrogen-cycling functional genes among different alfalfa stand ages during the maturity-harvest stage. CK, maize-field control without alfalfa cultivation; T1, 2-year-old alfalfa field; T2, 3-year-old alfalfa field; T3, 4-year-old alfalfa field; T4, 7-year-old alfalfa field. Gene abundance values represent metagenome-derived TPM-normalized relative abundances rather than absolute gene copy numbers. Data are presented as means ± SE (*n* = 3). Different lowercase letters indicate significant differences among treatments at *p* < 0.05. ns indicates no significant difference (*p* ≥ 0.05), whereas * and *** indicate significance at *p* < 0.05 and *p* < 0.001, respectively, whereas ns indicates no significant difference (*p* < 0.05).

Across the three growth stages, nitrogen-cycling functional genes displayed distinct temporal variation patterns. Overall, most functional genes showed relatively higher abundances during the regreening stage, particularly narG/nxrA, napA, nirS, nirB, and nasA. During the flowering stage, differences among cultivation treatments became more pronounced, with multiple functional genes exhibiting significant or highly significant variation. During the maturity-harvest stage, the abundances of some functional genes decreased, whereas nirK, nirS, and norB maintained relatively higher abundances in the T3 treatment. The temporal patterns differed among functional genes; for example, amoA showed relatively high abundance during the regreening and flowering stages but declined during the maturity-harvest stage, whereas hao exhibited no significant differences among treatments at the maturity-harvest stage. Overall, growth stage was significantly associated with the abundance patterns of soil nitrogen-cycling functional genes, and different functional genes exhibited distinct responses to alfalfa cultivation duration and growth stage.

### 3.3 Correlations between nitrogen-cycling functional genes and soil inorganic nitrogen

As shown in Figure 6, the correlations between nitrogen-cycling functional genes and soil inorganic nitrogen concentrations varied substantially across different growth stages. During the regreening stage, most nitrogen-cycling functional genes showed negative correlations with both ${\text{NO}}_{3}^{-}$-N and ${\text{NO}}_{4}^{+}$-N. Specifically, hao exhibited significant negative correlations with both ${\text{NO}}_{3}^{-}$-N and ${\text{NO}}_{4}^{+}$-N, while nirS showed significant and highly significant negative correlations with ${\text{NO}}_{3}^{-}$-N and ${\text{NO}}_{4}^{+}$-N, respectively. In addition, narG/nxrA, nirB, and nasA were significantly negatively correlated with ${\text{NO}}_{4}^{+}$-N.

**Figure 6:**
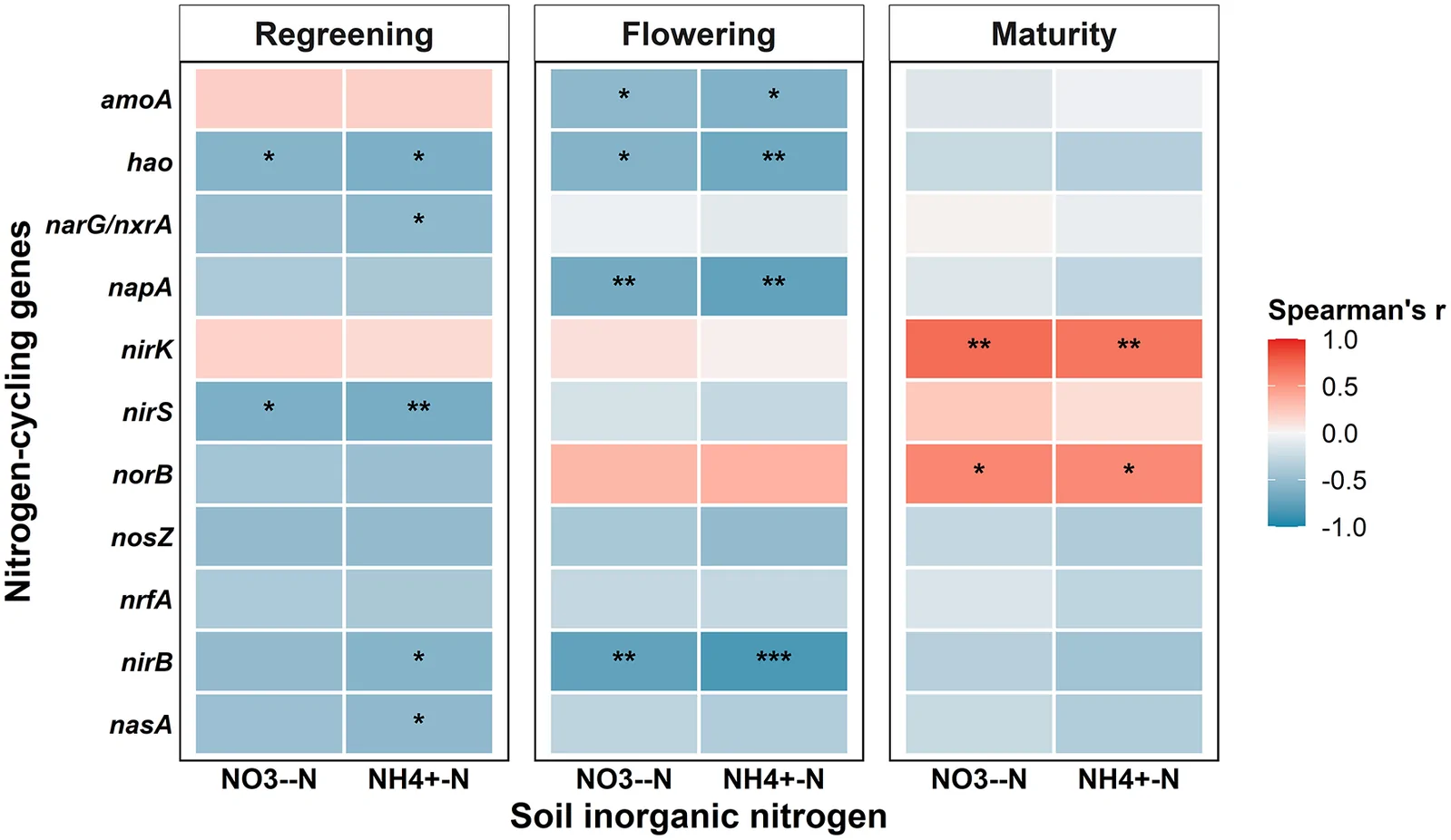
Spearman correlation heatmap showing the relationships between nitrogen-cycling functional genes and soil inorganic nitrogen concentrations. Color gradients represent Spearman correlation coefficients, with red indicating positive correlations and blue indicating negative correlations. *, **, and *** indicate significance levels at *p* < 0.05, *p* < 0.01, and *p* < 0.001, respectively.

During the flowering stage, negative correlations remained predominant among most functional genes. The abundances of amoA, hao, napA, and nirB showed significant or highly significant negative correlations with ${\text{NO}}_{3}^{-}$-N and ${\text{NO}}_{4}^{+}$-N. In contrast, nirK and norB exhibited positive correlation trends with soil inorganic nitrogen, although these correlations were not statistically significant.

During the maturity-harvest stage, several denitrification-related genes showed significant positive correlations with soil inorganic nitrogen. Specifically, nirK exhibited highly significant positive correlations with both ${\text{NO}}_{3}^{-}$-N and ${\text{NO}}_{4}^{+}$-N, whereas norB showed significant positive correlations with both nitrogen forms.

Overall, the associations between nitrogen-cycling functional genes and soil inorganic nitrogen exhibited clear growth-stage dependence. Negative correlations dominated during the regreening and flowering stages, whereas several denitrification-related genes showed positive correlations with soil inorganic nitrogen during the maturity-harvest stage.

### 3.4 Exploratory structural equation modeling of associations among nitrogen-cycling functional genes and soil inorganic nitrogen dynamics

The exploratory structural equation model (SEM) was used to examine the associations among alfalfa stand age, growth stage, nitrogen-cycling functional gene groups, and soil inorganic nitrogen (Figure 7). The model did not show satisfactory global fit (χ^2^ = 92.325, df = 3, *p* < 0.001, CFI = 0.656, TLI = −0.605, RMSEA = 0.813, SRMR = 0.084). Therefore, the SEM results were interpreted cautiously as exploratory statistical associations rather than confirmatory evidence of causal relationships.

**Figure 7:**
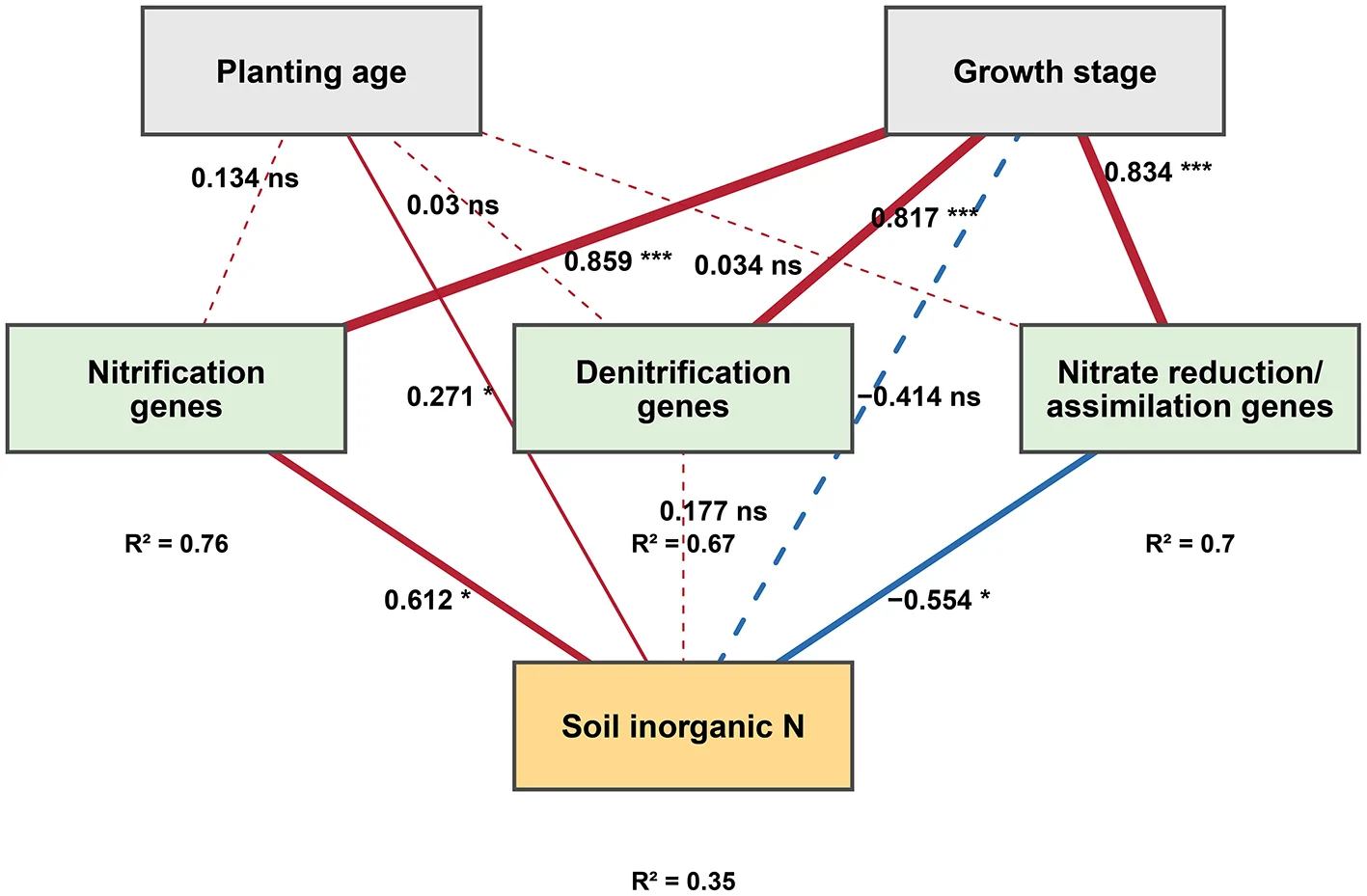
Exploratory structural equation model showing statistical associations among alfalfa stand age, growth stage, nitrogen-cycling functional gene groups, and soil inorganic nitrogen. Red and blue arrows indicate positive and negative associations, respectively. Solid and dashed arrows represent significant and non-significant paths, respectively. Values beside the arrows indicate standardized path coefficients. R^2^ values indicate the proportions of variance explained for endogenous variables. The nitrate reduction/assimilation-related gene composite included napA, nirB, nrfA, and nasA, whereas narG/nxrA was excluded because it represents a nitrate/nitrite-transformation-related homolog group. *ns* indicates a non-significant association; * and *** indicate significance at *p* < 0.05 and *p* < 0.001, respectively. Given the observational chronosequence design and limited model fit, the paths represent exploratory statistical associations rather than confirmed causal relationships.

Growth stage showed significant positive associations with nitrification-related genes, denitrification-related genes, and nitrate reduction/assimilation-related genes, with standardized path coefficients of 0.859, 0.817, and 0.834, respectively. In contrast, alfalfa stand age was not significantly associated with any of these three functional gene groups. Alfalfa stand age showed a significant positive association with soil inorganic N (β = 0.271, *p* = 0.029), whereas the direct association between growth stage and soil inorganic N was not significant (β = −0.414, *p* = 0.231).

Regarding the associations between functional gene groups and soil inorganic N, nitrification-related genes showed a significant positive association with soil inorganic N (β = 0.612, *p* = 0.012), whereas nitrate reduction/assimilation-related genes showed a significant negative association with soil inorganic N (β = −0.554, *p* = 0.011). The association between denitrification-related genes and soil inorganic N was not statistically significant (β = 0.177, *p* = 0.395). The exploratory SEM explained 76%, 67%, 70%, and 35% of the variation in nitrification-related genes, denitrification-related genes, nitrate reduction/assimilation-related genes, and soil inorganic N, respectively.

Overall, the exploratory SEM suggested that growth stage was more strongly associated with variation in the three functional gene groups than alfalfa stand age in the present dataset. Nitrification-related genes and nitrate reduction/assimilation-related genes were statistically associated with soil inorganic N variation. However, given the limited global model fit, the observational chronosequence design, and the use of metagenome-derived functional gene abundances as indicators of functional potential, these relationships should be interpreted as exploratory associations rather than evidence of direct causal mechanisms.

## 4 Discussion

### 4.1 Effects of different durations of alfalfa cultivation on soil inorganic nitrogen concentrations

The results of this study demonstrated that different durations of alfalfa cultivation significantly affected soil ${\text{NO}}_{3}^{-}$-N and ${\text{NO}}_{4}^{+}$-N concentrations, with both nitrogen forms exhibiting a consistent pattern of initial increase followed by decline across the three growth stages. The highest concentrations were observed in the T3 treatment, whereas the T4 treatment showed decreased inorganic nitrogen levels compared with T3. These findings suggest that moderate-duration alfalfa cultivation is beneficial for soil inorganic nitrogen accumulation, but this positive effect does not continuously increase with prolonged cultivation duration. As a perennial leguminous forage crop, alfalfa can enhance soil nitrogen inputs and organic substrate availability through biological nitrogen fixation by root nodules, rhizodeposition, root turnover, and residue return. These processes provide substrates for soil nitrogen mineralization and subsequent nitrification, thereby contributing to the accumulation of ${\text{NO}}_{4}^{+}$-N and ${\text{NO}}_{3}^{-}$-N (Mei et al., 2024; Xu et al., 2023). Recent studies have also demonstrated that alfalfa cultivation duration significantly influences soil mineral nitrogen, nutrient availability, and microbial community composition, with these effects generally exhibiting strong duration dependence (Zhang et al., 2023; Qin et al., 2024).

Among different cultivation durations, the relatively lower inorganic nitrogen concentrations observed in the T1 and T2 treatments may be attributed to the relatively short establishment period of alfalfa, during which root development and biological nitrogen fixation effects had not yet fully developed. The relatively high values observed in the T3 treatment may be related to differences in root-derived inputs and residue inputs, which could contribute to a greater potential for organic nitrogen mineralization and inorganic nitrogen accumulation. However, root activity, belowground biomass production, and nitrogen mineralization rates were not directly measured in this study, and this interpretation therefore remains speculative. However, the decline in ${\text{NO}}_{3}^{-}$-N and ${\text{NO}}_{4}^{+}$-N concentrations in the T4 treatment compared with T3 suggests that the positive effect of alfalfa cultivation may weaken in the 7-year-old stand. This reduction may be associated with increased nutrient export caused by repeated harvesting, reduced plant growth vigor, decreased root activity, and limited availability of soil organic substrates. Studies on aging alfalfa fields have indicated that long-term continuous cultivation may induce negative succession of rhizosphere microbial communities and accumulation of autotoxic metabolites, thereby affecting plant growth, rhizosphere environments, and soil nutrient retention capacity (Ma et al., 2024a,b). Therefore, the decline in inorganic nitrogen concentrations observed in the T4 treatment may result from the combined effects of repeated nutrient removal, rhizosphere ecological changes, and alterations in nitrogen transformation processes. Previous studies have similarly suggested that early- to medium-term alfalfa cultivation generally promotes soil nutrient accumulation, whereas soil improvement effects may decline under prolonged continuous cultivation (Ali et al., 2023; Li et al., 2018; Wang et al., 2024; Liu et al., 2025). This interpretation is further supported by recent synthesis and meta-analysis studies. Ali et al. (2023) reported that soil carbon and nitrogen responses in alfalfa pasture were closely associated with stand age, and that the improvement effects did not increase indefinitely with increasing stand age. Li Y. et al. (2024) also showed, based on a meta-analysis, that planting years significantly affected alfalfa yield and quality, indicating that the ecological and production effects of alfalfa are strongly stand-age dependent. These findings are consistent with the present observation that soil inorganic nitrogen increased from the early alfalfa stands to the 4-year-old stand but declined in the 7-year-old stand.

Across different growth stages, ${\text{NO}}_{3}^{-}$-N concentrations remained relatively high during the flowering and maturity-harvest stages, whereas ${\text{NO}}_{4}^{+}$-N concentrations were relatively higher during the regreening stage. These temporal patterns may be associated with variations in plant nitrogen uptake intensity, soil temperature and moisture conditions, seasonal precipitation, and microbially mediated nitrogen transformation processes during alfalfa growth. In the study area, precipitation was concentrated during summer, and the flowering stage occurred in July, when rainfall was relatively high. Increased rainfall during this period may enhance soil water movement and promote the leaching or vertical redistribution of inorganic nitrogen, particularly ${\text{NO}}_{3}^{-}$-N, which is more mobile than ${\text{NO}}_{4}^{+}$-N. Therefore, changes in soil inorganic nitrogen during the flowering stage may reflect the combined effects of plant nitrogen uptake, microbial nitrogen transformation, and rainfall-induced nitrogen leaching. During the regreening stage, rapid recovery of alfalfa growth may enhance soil organic nitrogen mineralization and promote ${\text{NO}}_{4}^{+}$-N accumulation. In contrast, during the flowering and maturity-harvest stages, increased temperature and microbial activity may accelerate the conversion of ${\text{NO}}_{4}^{+}$-N to ${\text{NO}}_{3}^{-}$-N through nitrification, thereby maintaining relatively higher ${\text{NO}}_{3}^{-}$-N concentrations. Wan et al. (2025) also reported that nitrogen uptake, biological nitrogen fixation, and soil inorganic nitrogen availability varied among alfalfa growth stages under different nitrogen supply conditions. Overall, the T3 treatment was more favorable for maintaining soil inorganic nitrogen accumulation, whereas the decline observed in T4 indicates that moderate stand age may help sustain soil nitrogen availability. However, management of alfalfa fields with different stand ages requires consideration of nutrient replenishment, biomass removal through harvesting, precipitation-driven nitrogen redistribution, and rhizosphere microbial processes (Gu et al., 2023).

### 4.2 Effects of alfalfa cultivation duration on nitrogen-cycling functional gene abundances

Nitrogen-cycling functional genes represent the functional potential of soil microorganisms involved in nitrogen transformation processes. In this study, different durations of alfalfa cultivation significantly altered the abundances of functional genes associated with nitrification, denitrification, and nitrate reduction/assimilation, with pronounced differences among growth stages. Overall, most nitrogen-cycling functional genes exhibited relatively higher abundances during the regreening stage and declined thereafter. However, differences among cultivation durations became more evident for several denitrification- and nitrate reduction/assimilation-related genes during the flowering and maturity-harvest stages. These patterns suggest that the effects of alfalfa cultivation duration on nitrogen-cycling functional potential may not follow a simple linear accumulation pattern and may be related to plant growth stage, soil inorganic nitrogen availability, rhizosphere substrate inputs, and microbial community composition (Li Q. P. et al., 2024; Xu et al., 2023; Qin et al., 2024). Consistent with this interpretation, PERMANOVA showed that growth stage explained most of the variation in nitrogen-cycling functional gene profiles (marginal R^2^ = 0.961, *p* = 0.001), whereas alfalfa stand age explained a much smaller but significant proportion of the variation (marginal R^2^ = 0.017, *p* = 0.002). These results suggest that seasonal plant development was more strongly associated with variation in nitrogen-cycling functional gene profiles than stand age in the present dataset. Among nitrification-related genes, amoA showed the strongest response to cultivation duration during the regreening stage, with significantly higher abundance in the T3 treatment than in the other treatments. This finding suggests that approximately 4 years of alfalfa cultivation may be more conducive to maintaining ammonia oxidation potential. The amoA gene encodes the ammonia monooxygenase enzyme, which is a key functional marker involved in ammonia oxidation, and its abundance is commonly associated with ammonium availability, microbial activity, and rhizosphere environmental conditions. In the present study, the higher amoA abundance observed in the T3 treatment was consistent with its higher soil ${\text{NO}}_{4}^{+}$-N, ${\text{NO}}_{3}^{-}$-N, microbial biomass nitrogen, and urease activity levels, suggesting that moderate cultivation duration may enhance nitrification-related functional potential by increasing substrate availability and microbial metabolic activity. Similarly, Li L. et al. (2025) reported that alfalfa cultivation duration influenced ammonia-oxidizing microbial communities by altering environmental factors such as soil moisture, total nitrogen, and ammonium nitrogen in alfalfa fields on the Loess Plateau, further indicating the sensitivity of amoA to changes associated with alfalfa cultivation duration.

Denitrification-related genes showed more pronounced differentiation among cultivation durations during the flowering and maturity-harvest stages. The significant variations in nirK, nirS, norB, and nosZ abundances among treatments indicate that alfalfa cultivation duration can influence denitrification functional potential. Previous studies have demonstrated that denitrification-related genes are highly responsive to changes in soil organic carbon, pH, nitrogen forms, and microbial community composition (Yang et al., 2024; Du et al., 2024). Sun et al. (2023) found that alfalfa cultivation duration significantly altered the community structures of nirK- and nirS-type denitrifying bacteria in alfalfa fields on the Loess Plateau, and that nirS abundance was closely associated with SOC, total nitrogen, and microbial biomass carbon. In the present study, relatively higher abundances of nirS and norB were observed in the T3 treatment during the maturity-harvest stage, which may be related to the higher inorganic nitrogen availability, greater rhizosphere substrate inputs, and enhanced microbial activity under this treatment. However, functional gene abundance represents microbial functional potential rather than actual process rates; therefore, further measurements of denitrification rates or gaseous nitrogen emissions are required to verify these processes.

Nitrate reduction- and assimilation-related genes, including napA, nasA, nrfA, and nirB, also exhibited stand-age-dependent variation across different growth stages, suggesting that alfalfa stand age may affect nitrate reduction, nitrate assimilation, and dissimilatory nitrate reduction processes in soils. These changes may be associated with differences in soil ${\text{NO}}_{3}^{-}$-N availability, plant nitrogen uptake, microbial nitrogen demand, and soil redox conditions among stand ages (Xu et al., 2023). In contrast, under the applied NCycDB annotation and TPM-based detection criteria, qualified nifH annotations were detected only in the regreening-stage samples, and no significant differences among treatments were observed. One possible explanation is that stronger early-season plant nitrogen demand and active root regrowth may have contributed to the detection of nifH during the regreening stage by influencing diazotrophic microorganisms in the rhizosphere. However, this explanation remains speculative because root activity, nodule abundance, nitrogenase activity, nifH transcript abundance, and plant nitrogen uptake were not directly measured in this study. In addition, root nodules were not separately collected or analyzed, and their possible contribution to the observed nifH pattern could not be evaluated separately. No qualified nifH annotation was detected in the flowering- or maturity-harvest-stage samples. This non-detection may have been related to the relative abundance of nifH, sequencing depth, or the applied annotation criteria, but these possibilities could not be directly evaluated in the present study. Therefore, the absence of a qualified nifH annotation should not be interpreted as evidence that nitrogen-fixing microorganisms or biological nitrogen fixation were completely absent during these stages. Rather, it indicates that nifH was not detected under the metagenomic sequencing and annotation conditions used in this study. This result does not imply that biological nitrogen fixation by alfalfa is unimportant; instead, it suggests that metagenomic profiling of rhizosphere soil alone may not fully capture nitrogen fixation associated with alfalfa. Yan et al. (2025) demonstrated that alfalfa cultivation influenced nitrogen-cycling functional groups, including nifH, ammonia-oxidizing microorganisms, nirK, nirS, norB, and nosZ, and that soil pH, electrical conductivity, SOC, and available nitrogen were important environmental factors shaping nitrogen-cycling microbial functions. Therefore, the contribution of alfalfa biological nitrogen fixation should be further evaluated by integrating qPCR-based nifH quantification, nodule abundance, nitrogenase activity, plant nitrogen accumulation, and soil nitrogen balance.

### 4.3 Pathways linking nitrogen-cycling functional genes to soil inorganic nitrogen dynamics

Correlation analysis revealed that the relationships between nitrogen-cycling functional genes and soil inorganic nitrogen varied markedly among growth stages. During the regreening and flowering stages, most functional genes exhibited negative correlations with ${\text{NO}}_{3}^{-}$-N and ${\text{NO}}_{4}^{+}$-N, whereas several denitrification-related genes, including nirK and norB, showed significant positive correlations with inorganic nitrogen during the maturity-harvest stage. These stage-dependent correlation patterns suggest that differences in plant nitrogen uptake intensity, soil substrate availability, and microbial nitrogen transformation processes among growth stages may contribute to the contrasting associations between functional genes and soil inorganic nitrogen. During the regreening stage, rapid recovery of alfalfa growth and increasing nitrogen demand may enhance microbial nitrogen transformation processes and plant nitrogen uptake, resulting in negative associations between functional gene abundances and residual soil inorganic nitrogen. During the maturity-harvest stage, reduced plant nitrogen uptake intensity and increased inputs of root residues and rhizosphere-derived substrates may enhance the availability of nitrogen substrates, potentially contributing to the increased abundance of some denitrification-related genes.

Previous studies have demonstrated that alfalfa cultivation can influence nitrogen-cycling microbial communities by altering soil nutrient conditions and microbial community composition, thereby affecting the abundances of nitrogen-cycling functional genes, including nifH, amoA, nirK, nirS, and nosZ (Zhou et al., 2021; Yang et al., 2024). Qin et al. (2024) and Yan et al. (2025) further suggested that changes in soil environmental conditions and microbial community succession during alfalfa establishment are important factors influencing nitrogen-cycling functions. Therefore, variations in rhizosphere substrate inputs and microhabitat conditions associated with different alfalfa growth stages may represent important drivers shaping nitrogen-cycling functional gene responses.

The exploratory SEM further suggested that growth stage was closely associated with variations in nitrogen-cycling functional gene groups and that some functional gene groups were statistically associated with soil inorganic nitrogen dynamics. However, because the SEM did not show satisfactory global model fit and was based on observational chronosequence data, these path relationships should be interpreted cautiously as exploratory statistical associations rather than direct evidence of causal mechanisms. Nitrification-related genes showed a significant positive association with soil inorganic N, suggesting that higher TPM-normalized abundances of nitrification-associated genes, such as amoA and hao, may reflect greater potential for ${\text{NO}}_{4}^{+}$-N transformation to ${\text{NO}}_{3}^{-}$-N. The nitrate reduction/assimilation-related gene group, which included napA, nirB, nrfA, and nasA, showed a significant negative association with soil inorganic N. This association may reflect the potential transformation or assimilation of nitrate into other nitrogen pools; however, it does not provide direct evidence of nitrate reduction activity. The narG/nxrA variable was not included in the nitrate reduction/assimilation-related composite because the K00370 annotation combines narG, narZ, and nxrA homologs associated with nitrate reductase or nitrite oxidoreductase functions. These homologs may participate in different nitrate/nitrite transformation processes, and their specific functional direction cannot be resolved from the available annotation. Accordingly, narG/nxrA abundance was interpreted as an indicator of potential nitrate/nitrite transformation rather than as a pathway-specific marker of nitrate reduction. Although previous studies have shown that napA is associated with nitrate reduction, nrfA-mediated dissimilatory nitrate reduction to ammonium (DNRA) can influence nitrogen retention in soils (Zhang et al., 2022; Saghaï et al., 2023), process-level measurements are still needed to verify these relationships in the present study.

Overall, soil inorganic nitrogen dynamics are unlikely to be explained by individual functional genes alone but may instead be associated with the combined responses of multiple nitrogen-cycling functional gene groups across growth stages. The observed association between moderate-duration alfalfa cultivation and nitrification-related functional potential may be related to differences in root-derived inputs, substrate availability, and microbial activity. Meanwhile, nitrate reduction/assimilation-, denitrification-, and narG/nxrA-related functional groups may be associated with changes in inorganic nitrogen pools through potential nitrogen transformation processes. However, functional gene abundance primarily represents microbial functional potential and cannot be directly interpreted as actual process rates, because nitrogen transformation processes are also influenced by gene expression, microbial activity, and enzyme efficiency (Zhang et al., 2013; Du et al., 2024). Therefore, future studies integrating measurements of nitrogen transformation rates, transcriptomic responses, enzyme activities, and gaseous nitrogen emissions are needed to further evaluate the contributions of nitrogen-cycling functional genes to soil inorganic nitrogen dynamics.

## 5 Conclusion

Different durations of alfalfa cultivation were significantly associated with changes in soil inorganic nitrogen concentrations and the abundances of nitrogen-cycling functional genes. Across the three growth stages, both ${\text{NO}}_{3}^{-}$-N and ${\text{NO}}_{4}^{+}$-N concentrations exhibited a unimodal response pattern, initially increasing and subsequently decreasing with increasing stand age. The highest inorganic nitrogen concentrations were observed in the T3 treatment, whereas the T4 treatment showed lower levels than T3. Thus, among the stand ages examined, the four-year alfalfa stand showed relatively favorable soil inorganic nitrogen levels under the specific soil, climatic, and management conditions of this study. However, this finding should not be interpreted as evidence that four years is universally optimal for alfalfa cultivation.

Nitrogen-cycling functional genes exhibited distinct response patterns across alfalfa stand ages and growth stages. Functional genes associated with nitrification, denitrification, and nitrate reduction or assimilation showed different temporal patterns among growth stages. PERMANOVA indicated that growth stage accounted for the largest proportion of variation in nitrogen-cycling functional gene profiles, whereas alfalfa stand age explained a significantly smaller proportion. Nitrification-related genes were positively associated with soil inorganic nitrogen, whereas the nitrate reduction/assimilation-related gene group was negatively associated with soil inorganic nitrogen, suggesting potential relationships with nitrogen transformation processes. The narG/nxrA variable was interpreted separately as a nitrate/nitrite-transformation-related homolog group rather than as a pathway-specific nitrate reduction marker.

Exploratory structural equation modeling further suggested that growth stage was strongly associated with variation in nitrogen-cycling functional gene groups. Nitrification-related genes showed a positive association with soil inorganic nitrogen, whereas the nitrate reduction/assimilation-related gene group showed a negative association. Because this study was based on observational chronosequence data, metagenome-derived functional gene abundances, and an exploratory SEM with limited global model fit, these relationships should be interpreted as statistical associations rather than direct evidence of causal mechanisms or actual nitrogen transformation rates. Further studies integrating process-level measurements, gene expression, nitrogen transformation rates, and plant nitrogen uptake are needed to verify the mechanisms underlying soil nitrogen dynamics in alfalfa fields of different stand ages.

## Data Availability

The datasets presented in this study can be found in online repositories. The names of the repository/repositories and accession number (s) can be found in the article/Supplementary material.
